# Recombinant ovine prion protein can be mutated at position 136 to improve its efficacy as an inhibitor of prion propagation

**DOI:** 10.1038/s41598-023-30202-0

**Published:** 2023-03-01

**Authors:** Katarzyna Kopycka, Ben C. Maddison, Kevin C. Gough

**Affiliations:** 1 School of Veterinary Medicine and Science, The University of Nottingham, College Rd., Sutton Bonington, Loughborough, LE12 5RD Leicestershire UK; 2 ADAS Biotechnology, Unit 27, Beeston Business Park, Technology Drive, Beeston, NG9 1LA Nottinghamshire UK

**Keywords:** Neuroscience, Diseases, Molecular medicine

## Abstract

Prion diseases are progressive neurodegenerative disorders with no effective therapeutics. The central event leading to the pathology in the diseases is the conversion of PrP^C^ into PrP^Sc^ and its accumulation in the central nervous system. Previous studies demonstrated that recombinant PrP (rPrP) and PrP peptides can inhibit the formation of PrP^Sc^. Here, the effectiveness of ovine rPrP mutants at codon 136 and peptides derived from this region were assessed for their ability to inhibit PrP^Sc^ replication, using protein misfolding cyclic amplification (PMCA). Based on a rPrP VRQ (rVRQ) genotype background (positions 136, 154 and 171) and mutations at position 136, the most effective inhibitors were V136R, V136K and V136P mutants, with IC50 values of 1 to 2 nM; activities much more potent than rVRQ (114 nM). rRRQ and rKRQ were also shown to effectively inhibit multiple ruminant prion amplification reactions that used distinct prion strain seeds and substrate *PRNP* genotypes. rRRQ, rKRQ and rPRQ were also shown to effectively protect Rov9 cells from scrapie infection when applied at 250 nM. The study demonstrates for the first time that the rPrP sequence can be mutated at sites known to be involved in prion disease susceptibility, to produce inhibitors with improved efficacy.

## Introduction

Prion diseases (transmissible spongiform encephalopathies, TSEs) are a group of fatal neurodegenerative conditions that are characterized by a protracted asymptomatic development phase. The central event in disease pathology is the conversion of the benign cellular prion protein (PrP^C^) into a distinct β-sheet rich conformation known as PrP^Sc^ that then accumulates^1^. In the central nervous system, PrP^Sc^ propagation and accumulation is accompanied by neuronal death, leading to clinical symptoms. The autocatalytic PrP^C^ to PrP^Sc^ conversion process is thought to proceed through a seeded polymerization mechanisms involving a PrP^C^-PrP^Sc^ complex^2^. Exactly how the PrP^C^ and PrP^Sc^ interact is not fully understood but it is thought that primary and secondary structures for PrP^C^ and PrP^Sc^ dictate whether such interaction can result in an efficient conversion of PrP^C^ to PrP^Sc^ and, as yet unknown, cofactors may be involved^3^. It is suggested that differences in the primary amino acid sequence of the PrP^C^ and PrP^Sc^ is a main driver for the so-called species barrier that largely prevents prion transmission between species^4–7^.

This group of diseases include Creutzfeldt Jakob Disease (CJD) in humans, scrapie in sheep and goats, bovine spongiform encephalopathy (BSE) in cattle and chronic wasting disease (CWD) in cervids. BSE caused an epidemic in the UK in the mid-1980s to 1990s and is a unique prion disease in that it crossed the species barrier, leading to BSE-like prion diseases in felines^1,8,9^, goats^10–13^ and humans. These cross-species transmission events were caused by ingestion of feed/food contaminated with BSE, and in humans the BSE-like disease is known as variant CJD (vCJD). It is estimated that 1 in 2000 individuals in the UK that were exposed at the time of the BSE epidemic may be carriers of vCJD prions^14^. These individuals are at risk of either developing the disease or being the source of iatrogenic transmission of the disease agent.

In humans, prion diseases can be genetic, acquired or spontaneous and the most common disease is sporadic CJD (sCJD) that annually affects approximately 1 to 2 people per million of the population^15,16^. At present, there are no effective therapeutics for these diseases despite considerable research effort. Potential therapeutics have included small molecules^17–20^, anti-PrP^C^/PrP^Sc^ antibodies^21–23^, and antisense oligonucleotides to lower PrP^C^ levels^24,25^. The development of therapeutics has used in vitro assays of prion replication such as protein misfolding assays^26–30^, cell models of infection^17,27,29,31–33^ and animal models of infection^34,35^. Several drugs have made it into human clinical trials. However, to date, no therapeutics have been shown to be effective in such trials (reviewed in^36^).

The development of effective therapeutics is further complicated by the existence and evolution/selection of prion strains. A prion strain is defined by a distinct pathology in a particular host and is often linked to measurable biochemical traits in the PrP^Sc^ molecule^37^. Multiple strains have been defined for most prion diseases and scrapie in sheep is particularly heterogeneous^38–42^. It is well established that one strain can shift to a distinct strain upon a change in the replication environment such as infection of a new host species or a host with a different *PRNP* genotype^43,44^. Furthermore, in vitro experiments have shown that under selective pressure of a drug that prevents prion replication, a new resistant PrP^Sc^ phenotype can emerge due to the evolution of a de novo PrP^Sc^ conformation or the selection of an existing minor population of a resistant PrP^Sc^ conformer^45^. This molecular plasticity may present challenges for the development of effective therapeutics as the prion may evolve in vivo to display resistance or a therapeutic against one prion strain may be ineffective against other strains.

An alternative approach to those mentioned above is to target the PrP^C^-PrP^Sc^ interaction by using a heterologous PrP^C^ molecule. It is well established that in in vitro and in animal models the presence of a heterologous PrP^C^ can block or delay disease or prion replication^26–30,35,46–48^. A further approach is to use recombinant PrP (rPrP) as the therapeutic and studies have shown that rPrP can prevent prion replication in in vitro assays^26–30^, in cell culture^27,29,46,47^ and also in rodent models^35,48^. Peptides of PrP have also been reported to be effective at inhibiting prion replication, including in cell culture models^26,27,29^. Within these studies, the use of peptides or the comparison of naturally occurring polymorphisms within PrP^C^ have identified residues or regions in PrP^C^ that may be involved in the conversion process, most likely, by binding to PrP^C^ substrate or PrP^Sc^^49^. Chabry and co-workers suggested the region 129–136 (hamster PrP) is critical for conversion which is a region equivalent to those containing polymorphisms linked with disease susceptibility in humans (human PrP residue 129) and sheep (sheep PrP residue 136)^26,27^. Residues 109, 112 and 139 in hamster PrP have also been suggested to be involved in the PrP^C^-PrP^Sc^ interaction^27^; and residues 138, 154 and 169 in murine PrP are suggested to be involved in the initial binding of PrP^C^-PrP^Sc^ but not in the conversion process itself^28^. In addition, residues 167, 171, 214 and 218 are dominant negative mutations for the conversion of wild type PrP^C^ to PrP^Sc^ if changed in PrP^C^ in a murine cell model of scrapie^50^. Ott and co-workers produced a small library of ~ 140 PrP variants that contained mutations in a single domain, they determined that changes Q167R, Q218K, S221P and Y217C in mouse PrP^C^ all acted as dominant negative mutants and protected cells from prion formation^51^. It has also been reported that both full length, as well as N-terminally and C-terminally truncated rPrP all bind effectively to PrP^Sc^, but only the full length version demonstrated maximal inhibition of conversion^29^. Similarly, mouse rPrP bound to human PrP^Sc^ but caused significantly less inhibition than human rPrP^29^. Overall, there is agreement that binding to PrP^Sc^ and inhibition of conversion are distinct processes involving sites across the inhibiting PrP molecule, and likely involve different binding sites on the PrP^Sc^ molecules^29^.

Here, we determined whether mutations at an amino acid residue in ovine PrP that is crucial in dictating susceptibility to scrapie, and therefore likely to influence PrP^C^-PrP^Sc^ interaction, can provide a mutant rPrP that is more effective at blocking prion replication than the natural variants previously reported^30^. The study used protein misfolding cyclic amplification (PMCA) as the main model of prion conversion. The method was developed by Soto and colleagues^52^ and has been adapted to ovine prion replication^53^. Our previous study demonstrated that VRQ (indicating amino acid polymorphisms at positions 136, 154 and 171, respectively) rPrP was the most effective inhibitor in this model when compared to natural ovine variants ARQ and ARR. Here, after screening all 20 variants at position 136 of ovine VRQ rPrP it was shown that several mutants consistently had lower IC50 values in PMCA compared to the natural VRQ variant. rPrP RRQ and KRQ were two of the most effective inhibitors and could effectively inhibit the replication of multiple prion strains found in ruminants. The rPrP RRQ, PRQ and KRQ proteins were also shown to be effective at preventing prion infection in an established cell culture model of ovine scrapie^57^.

## Results

### Screening of inhibition of prion replication by PMCA

Mutations were introduced into the VRQ version of rPrP and confirmed by Sanger sequencing. Versions of rPrP with amino acids mutations at position 136 were then expressed and purified yielding between 3 and 7.5 mg/L purified rPrP.

**Figure 1:**
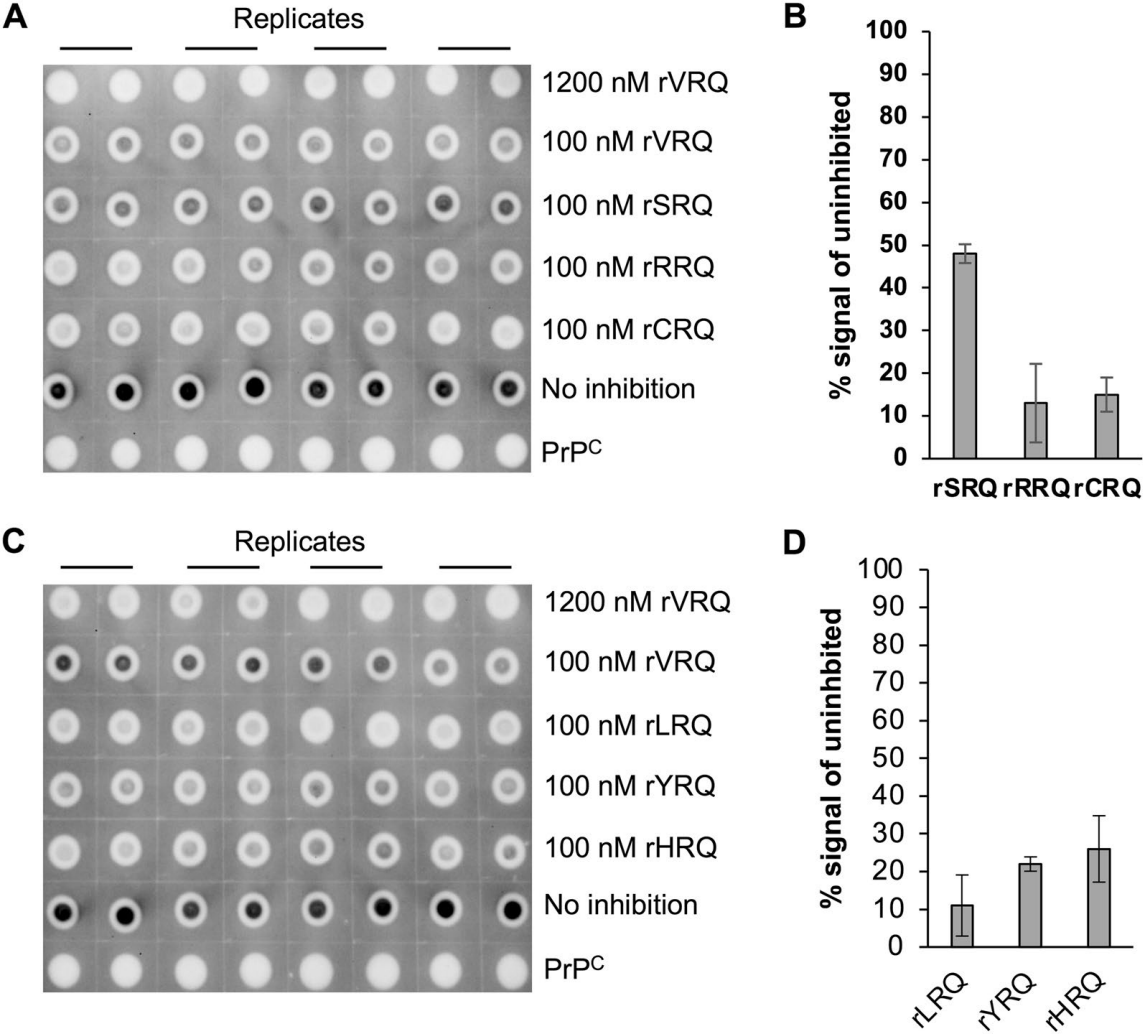
Inhibition of scrapie prion replication with 136 variants of rPrP. PMCA amplification of scrapie VRQ/ARQ prion in a VRQ/VRQ substrate was carried out in the absence or presence of a rPrP as indicated. Representative dot blot images are shown (**A**, **C**). Each sample was analysed in 8 replicate PMCA amplifications and 4 replicates were analysed (in duplicate) on each of two dot blots. Dot blots were analysed by densitometry and the PrP^Sc^ signals for each duplicate analysis were averaged. Then the signal above the blot background (1200 nM rVRQ inhibition) for each sample were expressed as a percentage of the signal for the no inhibition control (examples are shown in (**B**, **D**). Averages of the 8 PMCA replicates were used and SD is shown (**B**, **D**). PrP^C^ (brain PMCA substrate) was used as a PK-digestion control, inhibition with 100 nM rVRQ was also carried out in each experiment as a known inhibitor control.

**Figure 2:**
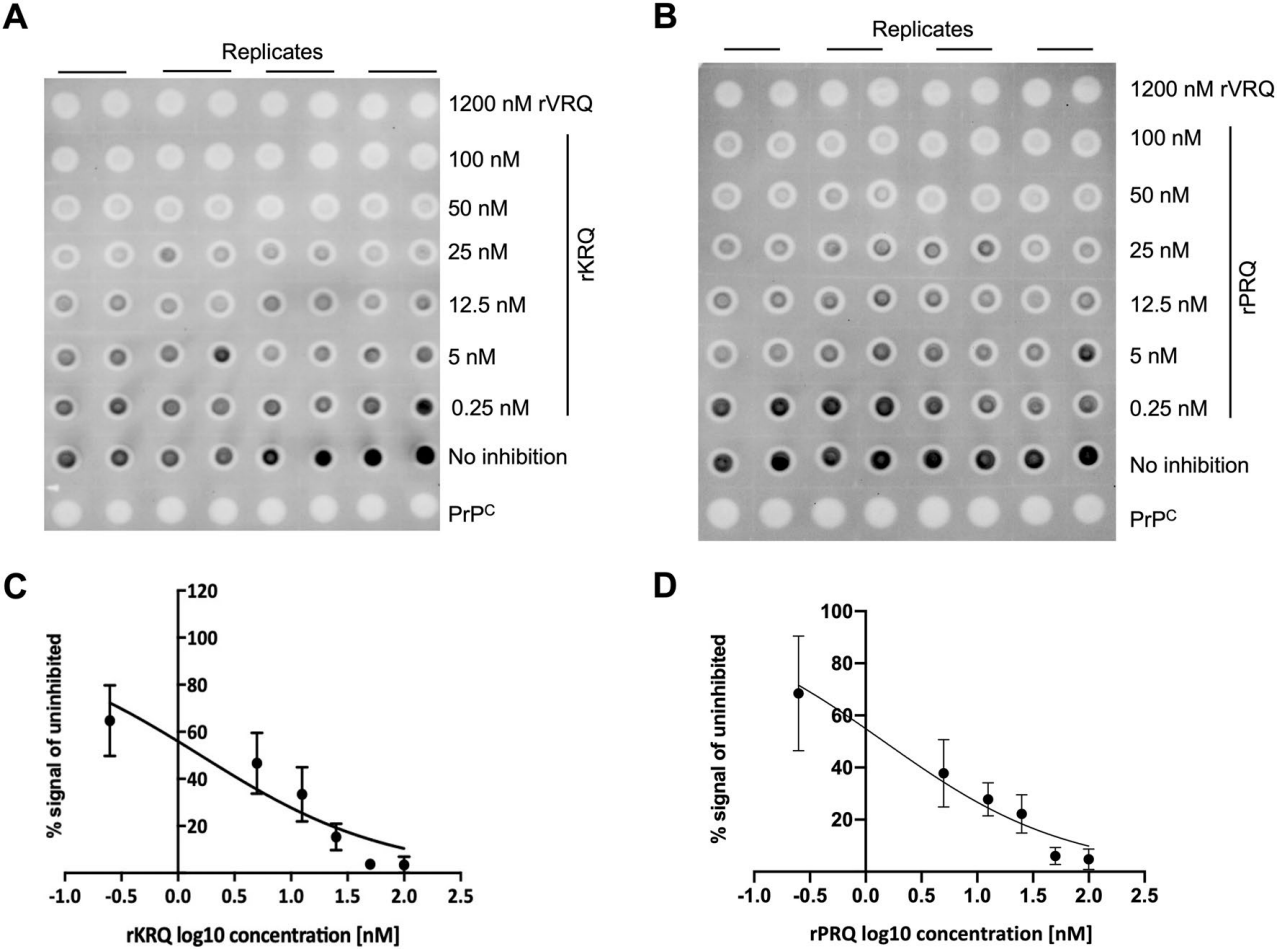
IC50 value determination for rPrP variants at position 136 inhibiting ovine scrapie replication. IC50 values were determined in the PMCA model of prion replication by measuring the level of inhibition for rPrP over 6 concentrations (as indicated for each rPrP). PMCA reactions were carried out in 4 replicates and each sample analysed in duplicate on dot blots (**A**, **B**). Dot blots were analysed by densitometry and the PrP^Sc^ signal above background (1200 nM rVRQ inhibition) were averaged for the duplicate analyses. The mean of replicate PMCA reactions with SD were plotted to calculate IC50 values (**C**, **D**). Representative examples are shown for rKRQ (**A**, **C**) and rPRQ (**B**, **D**).

All rPrP versions were assessed at 100 nM for their ability to inhibit the amplification of a VRQ/ARQ classical scrapie isolate in a single round of PMCA using VRQ ovine PrP^C^ substrate. 100 nM was chosen as this is close to the IC50 value for rVRQ which was previously reported as the most effective inhibitor of prion replication compared to the other natural variants ARQ and ARR^30^. Within 8 repeat experiments, the rVRQ inhibited amplification by $60 \pm 24\%$, as expected (data not shown). The average inhibition values for each of 18 further rPrPs (excluding rARQ) indicated that rRRQ, rCRQ, rLRQ, rYRQ, rHRQ, rKRQ, rPRQ and rERQ were the most effective, with inhibition levels of greater than 70% at 100 nM (Figs. 1 and S1, Table 1). To further quantify the level of inhibition for each rPrP, IC50 values were determined as previously described^30^. The rVRQ IC50 was estimated to be $114 \pm 25$ nM (from 3 repeat experiments, SI Figure S2 and data not shown) which corresponded well with the previously reported value of 122 nM^30^. The same concentration range used to estimate rVRQ efficacy, 50 to 1200 nM, was used for rRRQ, rCRQ, rLRQ, rYRQ and rHRQ (SI Figure S2). IC50s were produced for rCRQ and rHRQ of 112 (95%CI 83–155) and 69 nM (95%CI 36–150), respectively. For the other proteins, IC50s could not be determined as the lowest concentration of rPrP inhibited more than 50% of the PrP^Sc^ replication. It was noted that for rRRQ only, inhibition was 100% even at 50 nM (SI Figure S2), indicating this version of rPrP was the most effective mutant of those tested. Next, the rPrP concentration range 0.25 to 400 nM was used to re-assess inhibition for rRRQ and this produced an IC50 value of 2 nM (95%CI 0.7 to 3.1, SI Figure S2). The remaining selected rPrPs: rPRQ, rKRQ (both Fig. 2) and rERQ (SI Figure S2), were tested at a concentration range of 0.25 to 100 nM. This produced IC50 values for rKRQ and rPRQ of 1 (95%CI 0.8–2.1) and 2 (95%CI 0.8–2.3), respectively. No IC50 value could be obtained for rERQ as 100 nM failed to inhibit over 50% of replication (SI Figure S2). The IC50 was re-evaluated for rKRQ using the same rPrP concentration range and was found to be consistent (2 nM, 95%CI 0.9 to 2.6, SI Figure S2). Overall, the data clearly indicated that mutating residue 136 can produce mutants of rPrP that have improved efficacy compared to the best natural variant VRQ. In addition, the data demonstrated that rRRQ, rKRQ and rPRQ were the most effective inhibitors of scrapie prion replication.

**Table 1:** Inhibition of scrapie replication with 100 nM rPrP variants at position 136.

rPrP	% inhibition^1^	rPrP	% inhibition^1^	rPrP	% inhibition^1^
rIRQ	21	rLRQ	89	rQRQ	58
rFRQ	17	rYRQ	78	rPRQ	90
rNRQ	7	rHRQ	74	rTRQ	64
rSRQ	52	rKRQ	78	rDRQ	68
rRRQ	87	rGRQ	60	rERQ	96
rCRQ	85	rWRQ	37	rMRQ	53

^1^Dotblots of PMCA products were analysed by densitometry and the PrP^Sc^ signal above background (1200 nM rVRQ inhibition) were expressed as a percentage inhibition of the no inhibition control. Each sample was analysed in 8 replicate PMCA amplifications and the average % inhibition is shown. Analysis of inhibition versus a no inhibition control was carried out using a one-way ANOVA with Dunnett’s multiple comparison test for each rPrP, all rPrPs demonstrated significant inhibition except rFRQ (*p* = 0.12) and rNRQ (*p* = 0.89).

### Prion protein peptides as inhibitors of prion replication

**Figure 3:**
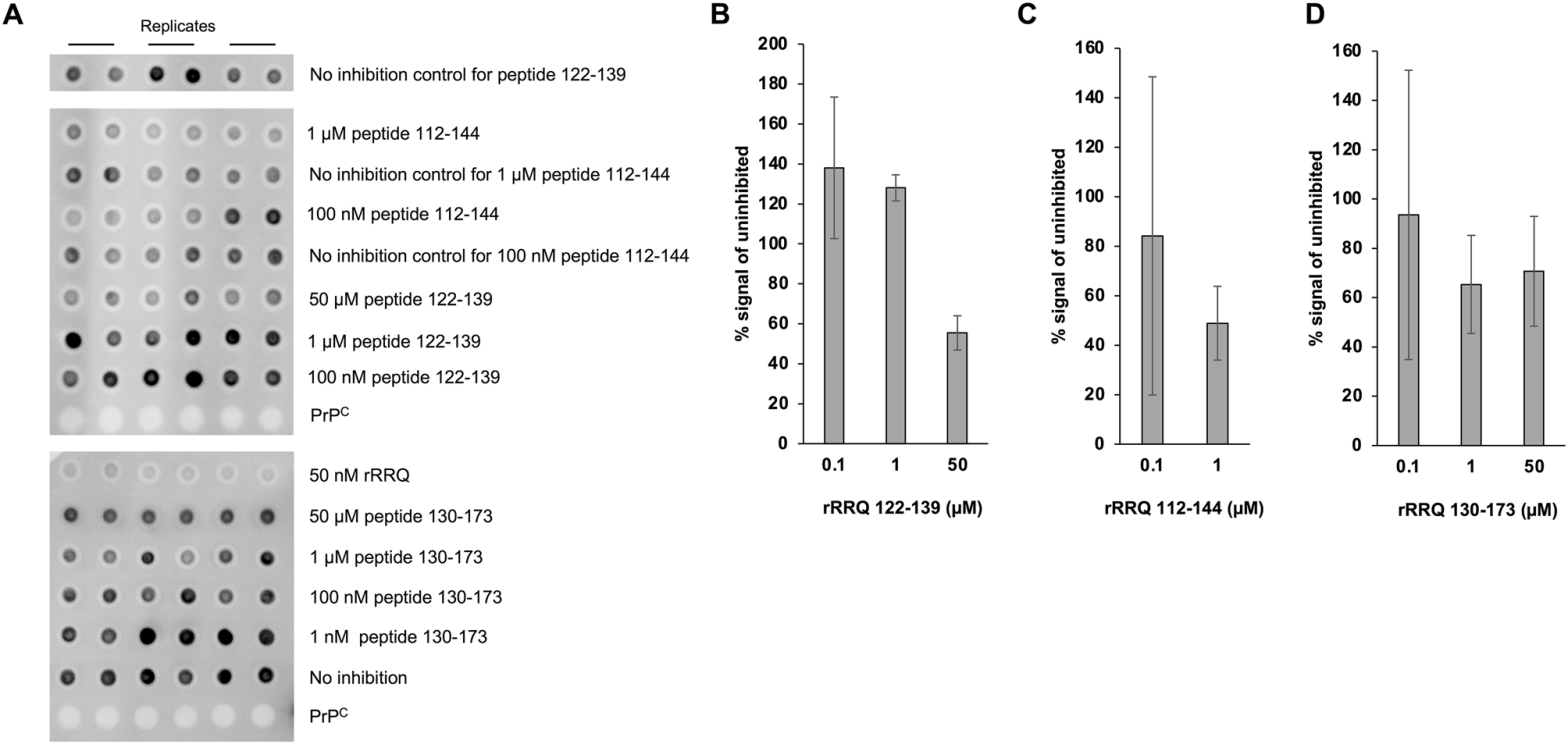
Prion replication inhibition with peptides of rRRQ spanning residue 136. PMCA amplification of scrapie VRQ/ARQ prion in a VRQ/VRQ substrate was carried out in the absence or presence of a rRRQ peptides (all in triplicate) and analysed by dot blot (each sample in duplicate). Peptides were composed of amino acid residues 122–139 (added at 100 nM to 50 μM, (**A** and **B**), 112–144 (100 nM or 1 μM, **A** and **C**) or 130–173 (1 nM to 50 μM as indicated, **A** and **D**). For peptide 112–144 the no inhibition control was carried out in the equivalent dilution of the peptide carrier solvent, 80% (v/v) acetonitrile. All other no inhibition controls were in PBS as the peptides were dissolved in water. Dot blot images (**A**), the top 2 panels are from the same blot (see Figure S3 for the full blot); the bottom panel is from a different blot) were analysed by densitometry to calculate the average signal compared to the corresponding no inhibition control (SD values are also shown for each PMCA triplicate; (**B**, **C**, **D**). Data was analysed using one-way ANOVA (with either Dunnett’s multiple comparison test for peptide 122–139 or 139–173, or Šidák’s multiple comparison test for peptide 112–139) at the concentrations used versus the relevant no inhibition control. No significant inhibition was seen for peptides at the concentrations tested. PrP^C^, brain PMCA substrate used as a PK-digestion control.

Having demonstrated that full length rPrP mutants could be effective inhibitors of prion replication and that mutations at residue 136 influenced the efficacy of inhibition, it was determined whether fragments of the rPrP containing the residue 136 could also be effective inhibitors. Fragments covering the amino acid residues 112–144 and 122–139 for rVRQ, rRRQ, rKRQ and rPRQ were produced and tested in the PMCA assay amplifying VRQ/ARQ scrapie. The 112–144 peptides did not show inhibition of prion replication at concentrations of up to 1 μM (Fig. 3 for RRQ peptides and Figure S3 for other peptides). Similarly, peptides 122–139 were tested at concentrations up to 50 μM and did not inhibit replication (Fig. 3 for RRQ peptides and Figure S3 for other peptides). A longer fragment covering residues 130–173 was then produced for rRRQ and again this fragment had no inhibitory effect on prion replication up to 50 μM (Fig. 3). No aggregation of peptides was observed upon re-suspension, although aggregation during the PMCA reactions cannot be discounted.

### Inhibition of the replication of multiple prion strains/isolates

**Figure 4:**
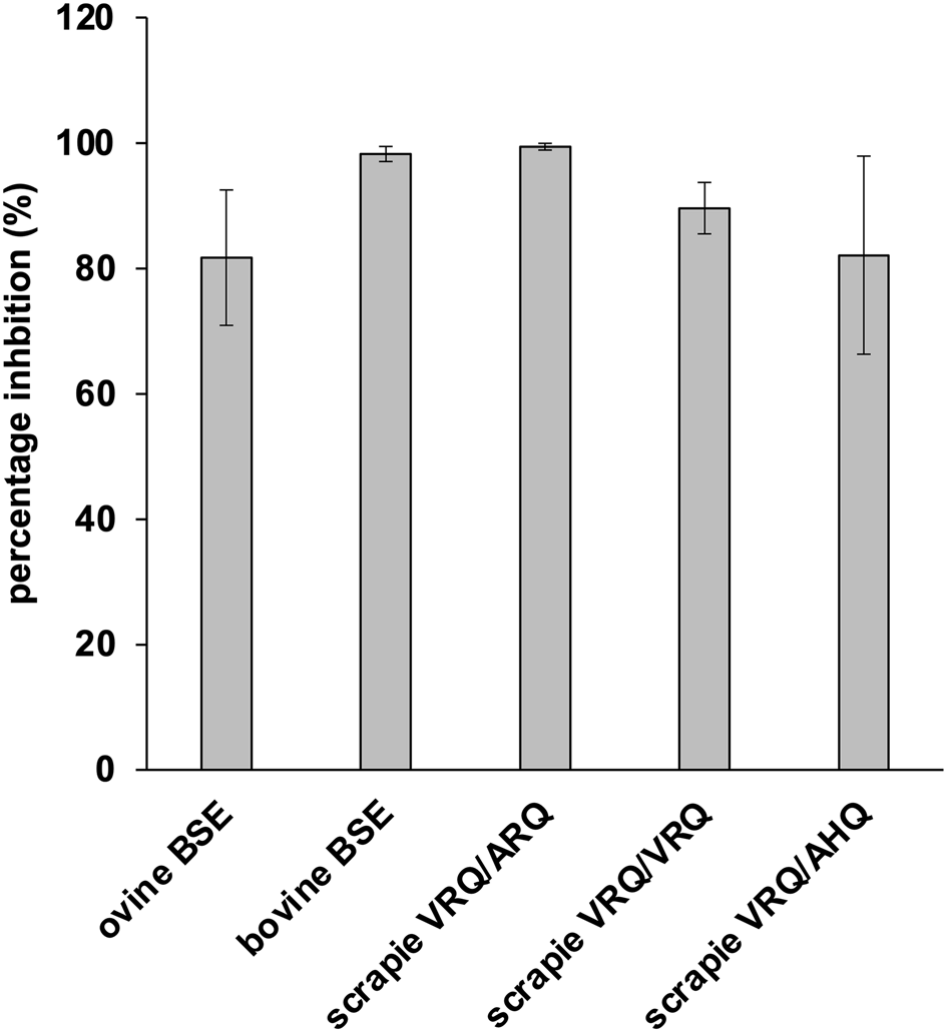
Inhibition of the replication of ruminant prion strains/isolates with rRRQ. Ovine BSE genotype ARQ/ARQ was amplified in 2 rounds using alternating ARQ/ARQ and AHQ/AHQ substrates. Bovine BSE was amplified in bovine brain substrate, and ovine scrapie isolates with VRQ/ARQ, VRQ/VRQ and VRQ/AHQ genotypes amplified in ovine VRQ/VRQ brain substrate; all amplified over 5 rounds of amplification. rRRQ was added into each round of PMCA at 50 nM. PMCA amplification in the absence of any rPrP was carried out as a no inhibition control. Dot blot images were analysed by densitometry and the PrP^Sc^ signal above background (samples with no PMCA amplification carried out in the final round of sPMCA) were expressed as a percentage of the no inhibition control. Three replicate PMCA reactions were carried out and each analysed in duplicate by dot blot, SD for the average signal for each of the triplicates are shown. rRRQ significantly inhibited each of the amplification reactions (+ /− inhibitor signals compared in a one-way ANOVA with Šidák’s multiple comparison test).

Any potential therapeutic would need to be effective against a range of prion variants. Serial PMCA (sPMCA) assays were used to amplify prions as they could not be amplified in a single PMCA round. Analysis was carried out at the sPMCA round that consistently amplified the prion strain under investigation. The most effective rPrPs at inhibiting amplification of a scrapie VRQ/ARQ isolate over a single round of PMCA, rRRQ, rPRQ and rKRQ, were tested in sPMCA for inhibition of other ruminant prion strains. This used 50 nM of rPrP added at each round of amplification. This was carried out for ovine BSE amplification over two rounds of sPMCA, and the amplification of bovine BSE and further ovine scrapie isolates (VRQ/VRQ and VRQ/AHQ genotypes, and VRQ/ARQ genotype for comparison) in sPMCA over 5 rounds (Fig. 4 and SI Figure S4). rRRQ was the most effective inhibitor, significantly reducing amplification for all prion strains/isolates tested. rKRQ significantly inhibited amplification of ovine BSE, VRQ/VRQ and VRQ/ARQ scrapie. rPRQ only significantly inhibited VRQ/ARQ scrapie amplification.

### Inhibition of prion infection of cells

**Figure 5:**
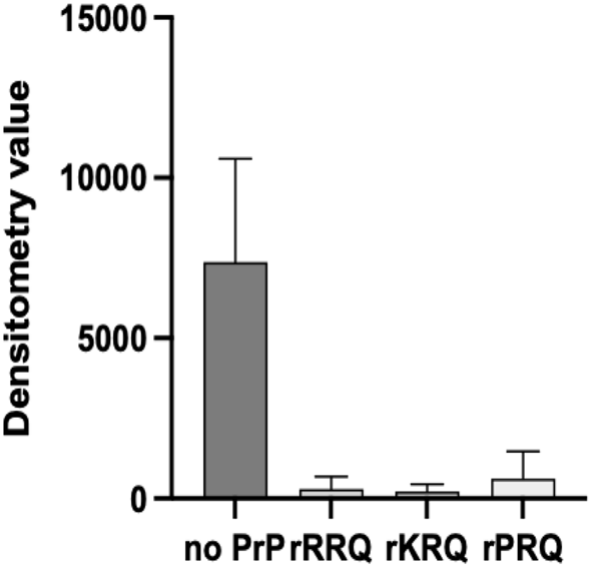
Inhibition of scrapie infection of cells with rPrPs. Rov9 cells expressing ovine VRQ/VRQ PrP^C^ were inoculated with SSBP/1 scrapie brain homogenate with or without the presence of 250 nM of rRRQ, rKRQ or rPRQ. After a single passage, cell lysate containing 500 µg of total protein was PK-digsted and analysed for PrP^Sc^ levels by western blot. Uninhibited cell infections were carried out in the absence of any rPrP as a control. The experiment was repeated three times for inhibition with rRRQ and twice for inhibition with rKRQ and rPRQ. Densitometry analysis of PrP^Sc^ signals were calculated and average signals with SD are shown. One-way ANOVA with Dunnett’s multiple comparison was applied and indicated significantly lower PrP^Sc^ signal for cells treated with 250 nM rRRQ, rKRQ and rPRQ compared to the no rPrP control cells.

Next, the effectiveness of rRRQ, rKRQ and rPRQ to prevent prion cell infections was tested. The model used Rov9 cells that express ovine VRQ/VRQ PrP^C^ and can be infected with the well characterized ovine scrapie strain SSBP/1^40^. rPrP was added to the SSBP/1 inoculum at 50 and 250 nM and then added to cells, following a single passage of cells, the levels of PrP^Sc^ were measured (Figs. 5 and S5). All three rPrPs were effective at reducing cell infections at 250 nM. rRRQ at 50 nM failed to prevent prion infection (SI Figure S5).

## Discussion

Both in vitro and in vivo studies have demonstrated that the presence of heterologous PrP^C^ can interfere with the conversion of homologous PrP^C^ to PrP^Sc^^26–30,35^, and the inhibiting PrP^C^ can differ from the homologous PrP^C^ by just a single amino acid residue^26,27,29,30,46^. These studies raise the possibility that heterologous PrP^C^ could be a potential therapeutic agent capable of blocking prion disease^48^. Cell free conversion assays^26–28^, PMCA^29,30^ and scrapie cell models^27,29,46,47^ have all been used to measure the ability of heterologous or homologous PrP^C^ or PrP peptides to block PrP^Sc^ propagation. rPrPs have also been shown to inhibit the conversion process. Yuan and co-workers demonstrated that homologous human rPrP could inhibit prion conversion in PMCA and this was more effective than heterologous mouse rPrP^29^. Furthermore, rVRQ inhibited propagation of PrP^Sc^ in PMCA for the conversion of substrate with a range of ovine PrP^C^ genotypes and bovine PrP^C^, for distinct ruminant prion seeds and where substrate/seed combinations had non-matching *PRNP* genotypes^30^. It has been suggested that rPrP may act as an effective inhibitor of PrP^Sc^ propagation as it lacks the GPI anchor and any glycosylation and so may be an ineffective conversion substrate^29^.

It has previously been shown that a natural polymorphism at position 136 of ovine PrP that strongly influences susceptibility to scrapie also had a significant effect on the inhibitory efficacy of ovine rPrP, with rVRQ and rARQ having IC50 values in PMCA of 122 nM and 288 nM, respectively^30^. The polymorphism at 171 also influenced this process and rARR had an IC50 of 505 nM^30^. As previous reports had also noted the region containing 136 as being critical in inhibiting replication^26,27^, the present study looked to introduce mutations into the 136 residue to engineer improved rPrP inhibitors. Eighteen distinct amino acids were introduced into this site in ovine rPrP (with the exceptions of V and A that had already been characterised^30^) and these were compared to rVRQ as inhibitors of scrapie replication in PMCA. An initial screen at 100 nM of inhibitor identified rRRQ, rCRQ, rLRQ, rYRQ, rHRQ, rKRQ, rPRQ and rERQ as being the most effective inhibitors of prion replication as each inhibited replication by more than 70%. rRRQ, rKRQ and rPRQ had the lowest IC50 values, 1 to 2 nM, indicating they were much more potent than rVRQ (IC50 value ~ 114 nM). rRRQ was further shown to effectively inhibit a range of ruminant prion PMCA reactions including with distinct prion strain seeds (scrapie isolates, bovine and ovine BSE) and substrate genotypes (ovine VRQ/VRQ, ARQ/ARQ and AHQ/AHQ, and bovine), with a mean of 89% inhibition at 50 nM. rKRQ inhibited amplification of ovine BSE, VRQ/VRQ and VRQ/ARQ scrapie, but rPRQ only inhibited VRQ/ARQ scrapie amplification. These data for rRRQ are in agreement with the previous report that rVRQ was an effective inhibitor across multiple TSE strains and PrP^C^ substrates^30^. The isolates/strains tested had distinct *PRNP* genotypes in both seed and substrate and whilst scrapie isolates could not be confirmed as distinct strains, the analysis of scrapie and BSE indicates rRRQ is capable of inhibiting multiple strains. Any potential therapeutic for prion diseases would need to be effective against as wide a range as possible of PrP^Sc^ conformers to not only be effective against multiple strains but also to help guard against the potential development of resistance. Here, rRRQ, rKRQ and rPRQ were also shown to effectively protect Rov9 cells from scrapie infection when applied at 250 nM. The efficacy of the mutant rPrPs described here compares favourably with other rPrPs reported previously. Human rPrP had an EC50 in PMCA of 60 nM and was the most effective inhibitor of human PrP^Sc^ replication when compared to murine, bovine and bank vole rPrPs, and truncated human rPrP (90–231 and 23–145). Human rPrP also inhibited prion replication in infected ScN2a cells at 100 and 1000 nM^29^.

Considering the stoichiometry of interaction of the rPrP with PrP^C^ and/or PrP^Sc^, Horiuchi and co-workers used a cell free conversion assay as a model for hamster and mouse PrP^Sc^ propagation being inhibited by homologous or heterologous PrP^C^^28^. They found that a 1:1 molarity of heterologous-PrP^C^ to substrate-PrP^C^ caused very high levels of inhibition. This occurred when these components were present at 50-fold less than the PrP^Sc^ seed molecules, indicating there are limited conversion sites on PrP^Sc^. Furthermore, both forms of PrP^C^ could bind the PrP^Sc^ and did not prevent the other from binding. It was also shown that homologous PrP^C^ in a high molar excess to the labelled PrP^C^ substrate did not reduce conversion of the latter, indicating that the PrP^C^ binding sites on PrP^Sc^ are not saturated. Together, these data indicate that the inhibiting PrP may bind and block conversion sites more favourably than substrate PrP^C^ and the latter can bind at other distinct site(s); and/or binding of inhibiting PrP causes conformational changes that prevents conversion of the substrate PrP^C^ to PrP^Sc^. Alternatively, the inhibiting PrP^C^ may bind more favourably than substrate PrP^C^ to a limited amount of cofactor, a process that does not affect interaction of either PrP^C^ with PrP^Sc^ but prevents conversion. Another possibility is that the inhibiting PrP may bind to the substrate PrP^C^ forming a complex that can bind PrP^Sc^ but cannot be effectively converted to PrP^Sc^. However, the latter is not supported in the study by Yuan and co-workers as rPrP was shown to bind PrP^Sc^ and not PrP^C^^29^. It is likely that the PrPs can bind PrP^Sc^ that contains a limited number of sites for PrP conversion, estimated to be 1 every 25 PrP^Sc^ molecules by Horiuchi and co-workers^28^. In the present study IC50 values of 1 nM indicate that 50% of the prion replication sites can be occupied/impaired by just 1 nM of inhibitor which is sub-stoichiometric amounts of rPrP compared to PrP^C^ (estimated to be 13 nM in 10% (w/v) brain^55^). This supports the above studies indicating that the inhibitory rPrPs bind more favourably to a crucial PrP^Sc^ site or cofactor compared to the substrate PrP^C^. Of further consideration on the mechanisms of inhibition is that not all PrP^C^ is converted to PrP^Sc^ and so only a fraction of the PrP^C^ substrate may be able to bind conversion sites/cofactors, this may enhance the competitiveness of rPrP blocking conversion.

Previously, peptides derived from the PrP sequence were shown to also inhibit PrP^Sc^ formation. Chabry and co-workers found that peptides 106–128, 113–141, 136–141 and 109–141 from hamster PrP inhibited PrP^Sc^ formation in a cell free conversion assay, although IC50 values were high at 230, 40, 68–75 and 30 μM, respectively^26,27^. Peptide 119–136 also inhibited cell free conversion of scrapie and reduced scrapie propagation in infected murine neuroblastoma cells with an IC50 of 11 μM^27^. They also reported that whilst PrP^C^ to PrP^Sc^ conversion was species specific when considering hamster and murine substrate and seed, the PrP peptides from hamster and mouse could inhibit PrP^Sc^ formation across species^27^. In addition, our previous study demonstrated that denatured rPrP could inhibit PrP^Sc^ replication, indicating that linear PrP interaction domains may be involved^30^. Here, the equivalent ovine peptides of the most effective hamster peptides described by Chabry et al.^26,27^ were produced: peptides 112–144 (hamster equivalent 109–141) and 122–139 (hamster equivalent 119–136) for rVRQ, rRRQ, rKRQ and rPRQ. None of the peptides significantly inhibited PrP^Sc^ formation in PMCA at 1 μM. Furthermore, the 122–139 peptide and the longer 130–173 peptide for rRRQ did not significantly inhibit PMCA at 50 μM. The data show that the PrP peptides were much less effective than full length rPrPs for inhibiting PrP^Sc^ formation. This data indicates that the interaction of the 136 PrP region is facilitated by the context in which it is held within the protein, for instance by stabilising this site and/or by additional contributing interaction sites elsewhere. The much shorter peptides cannot replicate this context. This supports the previously reported theory that this inhibition process involves multiple interaction domains across the inhibiting PrP molecule^29^.

In summary, previous studies using in vitro prion conversion assays and cell models of prion infection have presented data that show PrP^C^, rPrP and PrP peptides can all inhibit the formation of PrP^Sc^. Here, we establish that rPrP is a much more effective inhibitor than peptides derived from it. This study also demonstrates for the first time that mutating the rPrP sequence at sites known to be involved in prion disease susceptibility, likely representing sites that are either directly or indirectly involved in PrP^C^ interaction with PrP^Sc^, resulted in improved efficacy as inhibitors of PrP^Sc^ replication. The strategy produced rPrP inhibitors (rRRQ, rKRQ and rPRQ) with IC50 values of 1–2 nM. The rRRQ variant also demonstrated inhibition of PrP^Sc^ formation across distinct substrate and seed PrP sequences and prion strains, and all three variants prevented scrapie infection of susceptible cells. It seems likely that further iterative improvements of inhibitor performance can be achieved by targeting other similar sites within ovine PrP that represent natural polymorphisms influencing susceptibility or that have been established as sites of interaction with PrP^Sc^. At present, there are no established therapeutics for prion diseases and the presented strategy has the potential to produce highly effective inhibitors of prion replication that could be effective across species and prion strains.

Supporting such a strategy, a demonstration of PrP inhibitors in murine models of prion disease has been reported. A mouse model applying rPrP with the Q218K mutation (corresponds to human PrP-E219K) as a therapeutic demonstrated prolonged disease incubation time^29,56^. Similarly, a murine scrapie model was used to demonstrate that application of hamster rPrP could decreased pathology and the accumulation of PrP^Sc^, increased the time to clinical symptoms and extended survival times^35^. In both examples, rPrP was primarily delivered intracerebrally at the time of inoculation^35,56^. A possibly more efficient way to deliver rPrP would be by gene-based therapy, for example using lentivirus vectors. Toupet and co-workers used this strategy to express a dominant negative PrP^C^ in a scrapie mouse model^57^ where the therapy was delivered at late asymptomatic stages and reduced pathology and extended survival times^57^. The production of ever more effective rPrP inhibitors of prion replication coupled with advances in gene-therapy delivery may provide the building blocks for the development of an effective treatment for prion diseases.

## Methods

### Samples

Healthy and diseased ovine tissues (with the exception of SSBP/1) were obtained from the Animal and Plant Health Agency TSE-Archive (APHA, Addlestone, Surrey, UK). Classical scrapie infected brain material was obtained from scrapie positive specimens submitted to the APHA for testing. The ovine BSE sample originated from an ovine BSE challenge of sheep carried out by the APHA. The BSE positive bovine brain tissue was a pool of 71 BSE positive cases and was sourced from the APHA (SE1945/0035). 10% (w/v) brain homogenates were prepared as previously described^30^. Healthy ovine brain tissues were obtained from a scrapie-free, New Zealand derived flock (ARSU, APHA) and healthy bovine brain tissue from a confirmed BSE negative Fresian cow (ADAS Drayton, APHA). SSBP/1 was a gift from Professor Nora Hunter (Roslin Institute, Edinburgh, UK). All animal procedures at APHA and Roslin were performed under the Home Office (UK) and local ethical review committee’s approval and in compliance with the Animal (Scientific Procedures) Act 1986.

### Production of rPrP proteins

The *PRNP* gene for ovine PrP VRQ (23–231) was cloned into plasmid pET22b as previously described^30^. The codon at position 136 was mutated by inverse PCR. Non-overlapping phosphorylated primers were designed, so that the 5’ end of the forward primer starts at the mutation site, and the reverse primer is complimentary to the sequence before the mutation position. Primer sequences were 5’-ACTTCCCAGCATGTAGCCACC-3’ and 5’-NNNATGAGCAAGGCCTCTTATAC-3’ where NNN coded for the different amino acids at position 136. PCR (50 μl) reaction used 0.3 μM of each primer, 20 U/ml of Q5 polymerase (NEBL), Q5 high GC enhancer, Q5 reaction buffer, 0.4 μM dNTPs and 0.2 ng/μl plasmid template. PCR reactions were performed with initial denaturation at 95 °C for 5 min. Next, 30 cycles of denaturation at 95 °C for 30 s, annealing at 61 °C for 30 s and elongation at 72 °C for 5 min were completed. In the case of cloning in a codon for the Q residue at position 136, gradient inverse PCR was performed with annealing temperatures ranging from 50 to 63 °C. Lastly, the final extension was performed for 10 min at 72 °C. PCR amplicons of the expected size were gel purified using the Nucleospin Gel and PCR clean up kit following the manufacturer’s instructions (Macherey–Nagel). DNA template was removed by digestion with DpnI (NEBL) and the enzyme heat inactivated at 80 °C for 20 min. DNA was purified on the Nucleospin columns and then ligated in 50 μl reactions using 0.4 μl (200 U) T4 DNA ligase (NEBL) and 150 ng DNA with incubation at 16 °C for 16 h. Ligation reactions were then transformed into chemically competent DH3 *E. coli* NovaBlue cells (Novagen) following the manufacturer’s instructions. Single ampicillin-resistant colonies from agar plates were used to isolate DNA and the *PRNP* gene was Sanger sequenced using the T7 Forward primer.

rPrP proteins were expressed and purified exactly as previously described^30^. Briefly, recombinant proteins were expressed overnight at 37 °C, cells pelleted, freeze thawed and then lysed with lysozyme. After DNaseI treatment, washed cell-lysate pellets were then solubilised in 8 M urea. Soluble rPrP was then purified using FPLC using a IMAC Hi-Trap chelating column (charged with 0.1 M CuSO_4_) on an AKTA prime FPLC machine (GE Healthcare). Recombinant prion proteins were eluted using imidazole gradient (0–0.5 M) in 50 mM NaH_2_PO_4_, 300 mM NaCl, pH 7.5. Protein purity in eluate fractions was analysed by SDS-PAGE (NuPAGE™ 12% Bis–Tris gels, Invitrogen, with 1 × MOPS buffer) for 1 h at 200 W. Gels were stained with Instant Blue (Expedeon) and fractions with relatively pure rPrP were pooled together. Protein concentration and yield was determined for the pooled rPrP by Bradford assay against a BSA standard and samples stored at − 80 °C with 20% (w/v) sucrose. Before rPrP was used as an inhibitor in PMCA or cell infection experiments, imidazole and sucrose were removed by two rounds of dialysis against PBS (MW cut off of 10 kDa). The dialysed protein concentration was then determined by Bradford assay and purity shown to be between 71 and 96% (SI Figure S6).

### Production of PrP peptides

rPrP peptides (L-amino acids except Glycine) covering residues 112–144 and 122–139 were produced for rVRQ, rRRQ, rKRQ and rPRQ by Biomatik, and a peptide of residues 130–173 of the rRRQ variant was produced by Genecust; all peptides were at 40–60% purity. Peptides were resuspended in the buffer recommended by the manufacturer: 112–114 variants V, R, K and P were resuspended in 80% (v/v) acetonitrile; 122–139 variant V was resuspended in 18% (v/v) acetonitrile + 2% (v/v) formic acid; and 122–139 variants R, K and P and 130–173 variant R were resuspended in ultrapure water.

### PMCA as a model of prion replication

Protein misfolding cyclic amplification (PMCA) was used to amplify prions and assess any inhibitory properties of the rPrP or peptides of PrP. PMCA reactions were performed as previously described^30^. Each reaction was performed in clear 0.2 ml tubes (Sarstedt). Routinely, scrapie isolate PG1361/05 (VRQ/ARQ) was the source of the PrP^Sc^ seed, while 10% (w/v) healthy brain (VRQ/VRQ) was the source of PrP^C^ substrate. In single round PMCA reactions, 0.5 μL of 10% (w/v) scrapie PG1361/05 brain homogenate was added into each reaction in a final reaction volume of 100 μL. PMCA was performed for 24 h with repeat cycles of 40 s of sonication and 29 min 20 s incubation at 37 °C. Power was set to 180–200 W (S-4000 Misonix, Ultrasonic Liquid Processors). A range of concentrations of rPrP or PrP peptide were added to samples before the amplification started. For other TSE samples, no amplification was produced in a single PMCA round and so serial PMCA (sPMCA) experiments were carried out for between 2 and 5 rounds. Amplification and inhibition of PG1361/05 (VRQ/ARQ) was also carried out over 5 rounds for comparison. For sPMCA, 50 µL of reaction products from round 1 were added to 100 µL of fresh substrate (including appropriate inhibitors) and 100 µL of this was used in the subsequent round of PMCA. For sPMCA, PrP^Sc^ seeds were 0.5 µL of 10% (w/v) brain homogenate for PG1361/05 (VRQ/ARQ), PG1207/03 (VRQ/VRQ), and 5 µL of 10% (w/v) brain homogenate for ovine scrapie PG1499/02 (VRQ/AHQ), bovine BSE (SE1945/0035), ovine BSE (PG1693/03, ARQ/ARQ). Samples in the final sPMCA round were also directly frozen at − 20 °C without PMCA being carried out, these provided background signals on the blots for this round of amplification. All PMCA reaction products were stored at − 20 °C before analysis.

For the analysis of PrP peptides as inhibitors, it was first established whether the carrier solvent had any effects on PMCA (Figure S7). The presence of 80% (v/v) acetonitrile diluted to the equivalent of peptide at 50 μM in the PMCA reaction inhibited prion replication by > 60% and was not used further. The equivalent acetonitrile concentrations for 0.1 and 1 μM peptide reduced amplification by ~ 30%; for these concentrations of acetonitrile each PMCA reaction testing a peptide were compared to a PMCA reaction containing solvent at the equivalent concentration. The presence of 18% (v/v) acetonitrile + 2% (v/v) formic acid diluted to the same extent as tested peptide (up to 50 μM) had no effect on PMCA replication of scrapie prions. Where peptides were dissolved in water there was no effect on PMCA of the carrier solvent.

### Dot blot analysis

PMCA reaction products were processed and analysed by dot blot as previously described^30^ but with the following exception: samples were digested with 100 µg/mL proteinase K (PK), 0.045% (w/v) SDS for 1 h at 37 °C prior to analysis. PrP was detected with SHa31 monoclonal antibody (1:40,000), polyclonal goat anti-mouse horseradish peroxidase (HRP; 1:2000; Dako) and HRP substrate EZ-ECL (Biological Industries). Blots were then visualised using the ChemiDoc™ Imaging System (BioRad) and images analysed using ImageJ software. Background signals for each blot (as detailed in the results section) were subtracted from all other samples before analysis.

### rPrP-inhibition of scrapie cell infection

Rov9 containing the pTRE plasmid to express ovine PrP sequence (VRQ variant) was a gift from Dr Hubert Laude (Virologie et Immunologie Moléculaires, Jouy-En-Josas, France)^54^. Rov9 cells were seeded on 12 well plates at 1 × 10^5^ cells per well and grown at 37 °C in Eagle’s Minimum Essential Medium complete (EMEM, Sigma) for 48 h. Then, media was discarded and cells were washed with 1 × with Dulbecco Phosphate Buffered Saline (D-PBS) with MgCl_2_ and CaCl_2_ (D-PBS). OPTI MEM complete (5% FBS, 1% Pen/Strep/Glu, Gibco) with or without 1 µg/ml of doxycycline was added and cells were incubated for 48 h. Scrapie brain homogenate SSBP/1 (10% w/v) was heated at 80 °C for 20 min, and sonicated for 2 min (180–200 W) (4000 Misonix) at 37 °C. Treated brain homogenate was mixed with OPTI MEM media to a 1/80 dilution with or without 1 µg/ml doxycycline and with or without rPrP (at a final concentration of 50 or 250 nM). The diluted brain homogenate was then added onto the induced Rov9 cells (equivalent of 12.5 µl of 10% (w/v) brain homogenate per well). Cells were incubated for 72 h at 37 °C, 5% CO_2_. Media was then discarded and fresh OPTI MEM media complete with or without 1 µg/ml of doxycycline was added and cells incubated for 48 h. Media was discarded and cells were washed with 1 × D-PBS. Next, D-PBS was discarded and 500 µl of 1 × Trypsin/EDTA was added and cells were incubated for 5 min at 37 °C, 5% CO_2_. Cells were then collected into a sterile 1.5 ml tube and 500 µl of OPTI MEM media complete was added. Cells were washed 2 × with sterile PBS and then seeded into wells on 12-well plates in OPTI MEM complete with or without 1 µg/ml of doxycycline and grown for 7 days. Cells from each well were collected using 1 × Trypsin/EDTA and washed 2 × with D-PBS and lysed with 500 µl of lysis buffer (50 mM Tris, 0.5% (v/v) Triton X-100, 0.5% (w/v) sodium deoxycholate, pH 7.4) for 10 min on ice. Cell lysates were centrifuged for 2 min at 400 × g. Supernatants were collected and stored at − 20 °C for further analysis. Protein content of cell lysates was estimated using the BCA Protein Assay Kit (Pierce) following the manufacturer’s instructions.

### Western blot analysis

Cell lysate containing 500 µg of total protein was digested with 20 µg/ml PK and analysed by western blot. Samples were mixed with LDS sample buffer (Invitrogen) with 5% (v/v) β-mercaptoethanol and boiled at 100 °C for 10 min. Samples were resolved on NuPAGE™ 12% Bis–Tris gels (Invitrogen) with 1 × MOPS buffer for 1 h at 200 W. SeeBlue™ Plus2 Pre-Stained Protein Standard (Invitrogen) was used. Proteins were transferred onto polyvinylidene difluoride (PVDF) membrane. Transfer was performed for 75 min at 30 V in NuPAGE 1 × Transfer Buffer (Invitrogen). Membranes were blocked overnight in 5% (w/v) milk powder (SMA) in 1 × TBST at 4 °C. PrP was then detected and analysed as for dot blots.

### Statistical analysis

For all densitometry, calculated values were plotted using GraphPad Prism v9.0.1. Where multiple samples were compared to a single control, one-way ANOVA with Dunnett’s multiple comparison test was carried out. Where multiple samples were compared to their individual controls, one-way ANOVA with Šidák’s multiple comparison test was carried out. Statistical significance was assigned at a p value of 0.05. The absolute IC50 values were determined by nonlinear regression, log inhibitor verses normalised response (variable slope) model with constraints at 100% and 0%.

## Supplementary Information

Supplementary Information.

## Data Availability

All data supporting this study are included within the article and/or supporting information.
