# Supplementary material for: Recombinant ovine prion protein can be mutated at position 136 to improve its efficacy as an inhibitor of prion propagation
Source: Sci Rep. 2023 Mar 1;13:3452. doi: 10.1038/s41598-023-30202-0 (PMC9978027; doi:10.1038/s41598-023-30202-0)
Supplement: Supplementary file 1 — Supplementary Information. [file 41598_2023_30202_MOESM1_ESM.pdf]

Recombinant ovine prion protein can be mutated at position 136 to improve its efficacy as an inhibitor of prion propagation

Katarzyna Kopycka[1], Ben C. Maddison[2] and Kevin C Gough[1*#]

[1]School of Veterinary Medicine and Science, The University of Nottingham, College Rd., Sutton Bonington, Loughborough, Leicestershire, LE12 5RD. UK.

[2] ADAS Biotechnology, Unit 27, Beeston Business Park, Technology Drive, Beeston, Nottinghamshire, NG9 1LA, UK.

* Corresponding authors

[#] Kevin C. Gough http://orcid.org/0000- 0001-5211-2088

Further supporting data:

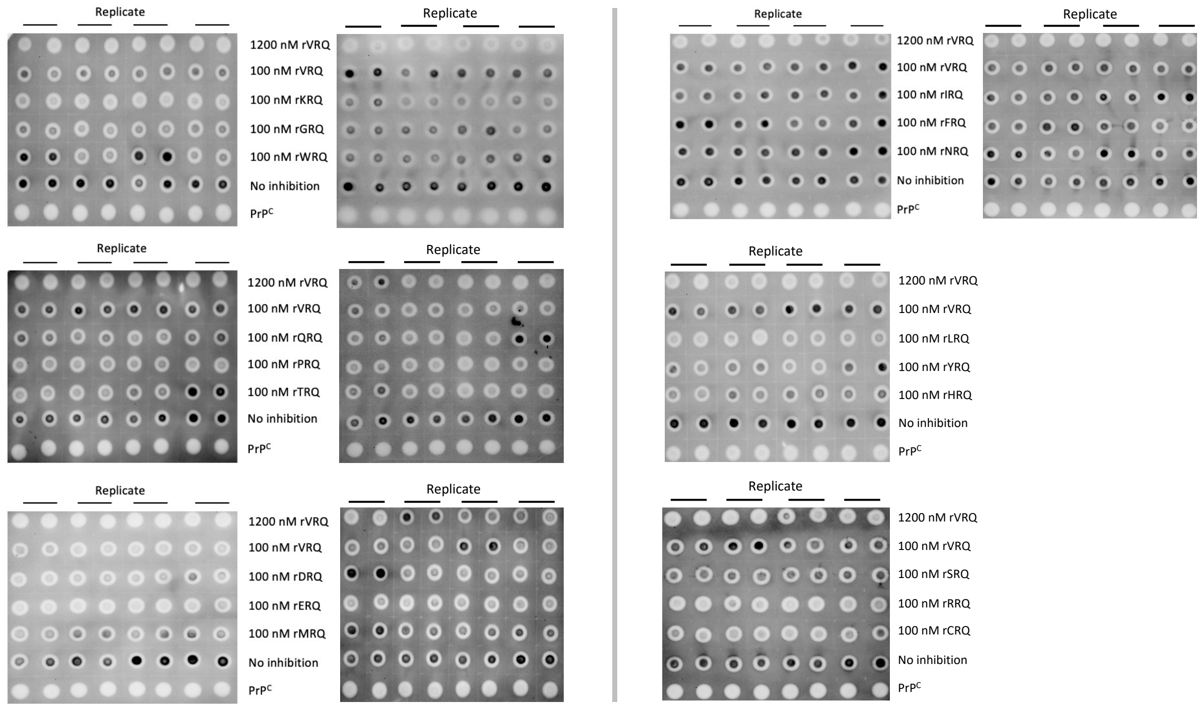

Figure S1. Inhibition of scrapie prion replication with 136 variants of rPrP. PMCA amplification of scrapie VRQ/ARQ prion in a VRQ/VRQ substrate was carried out in the absence or presence of a rPrP as indicated. Each sample was analysed in 8 replicate PMCA amplifications and 4 replicates were analysed (in duplicate) on each of two dot blots (also see Figure 1). Dot blots were analysed by densitometry and the PrP^{Sc} signals for each duplicate analysis were averaged. Then the signal above the blot background (1200 nM rVRQ inhibition) for each sample were expressed as a percentage of the signal for the no inhibition control. Averages of the 8 PMCA replicates were determined with SD. PrP^{C} (brain PMCA substrate) was used as a PK-digestion control, inhibition with 100 nM rVRQ was carried out in each experiment as a known inhibitor control.

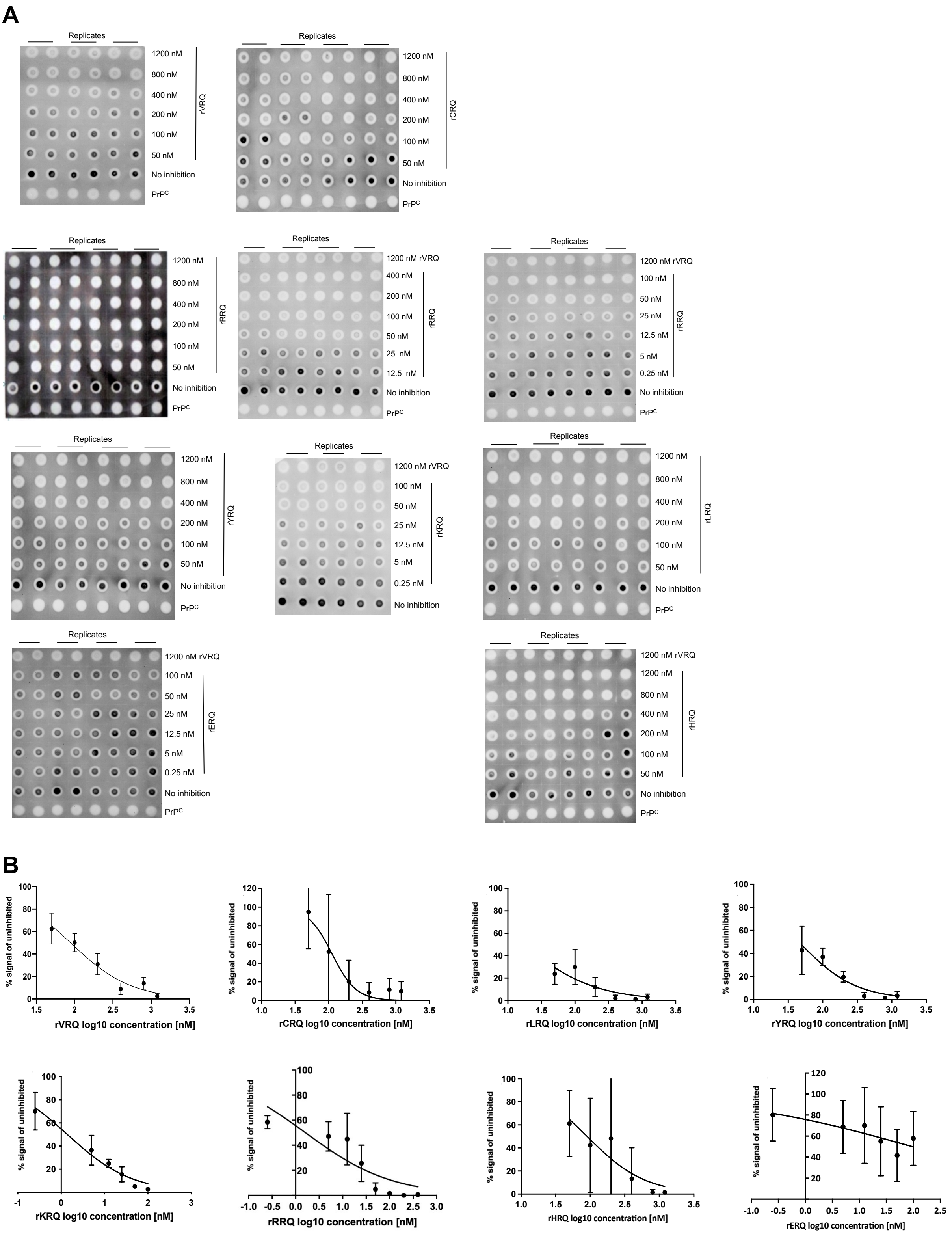

Figure S2. IC50 value determination for rPrP variants at position 136 inhibiting ovine scrapie replication. IC50 values were determined in the PMCA model of prion replication by measuring the level of inhibition for rPrP over a range of concentrations (as indicated for each rPrP on each blot). PMCA reactions were carried out in 3 or 4 replicates and each sample analysed in duplicate on dot blots (A). Dot blots were analysed by densitometry and the PrP^{Sc} signal above background were averaged for the duplicate analyses. The mean of

replicate PMCA reactions with SD were plotted to calculate IC50 values (B). N.B. for rRRQ, the concentration range 50 nM to 1200 nM completely inhibited PrP^{Sc} replication and data could not be plotted, concentration ranges of 12.5 to 400 nM and 0.25 to 100 nM were then analysed for this protein over 2 dot blots and the normalised data across these 8 concentrations used to calculate the IC50).

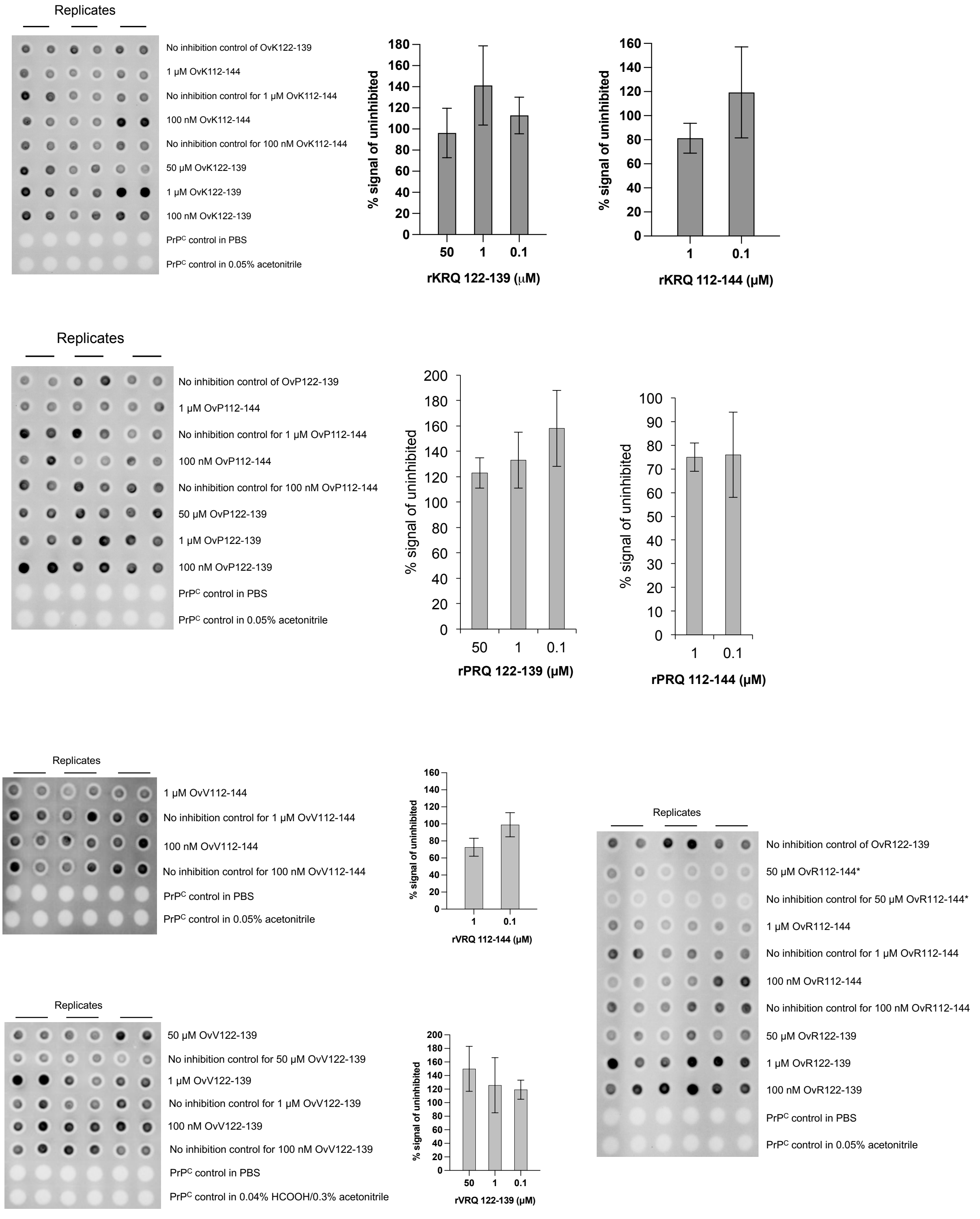

Figure S3. Prion replication inhibition with peptides of rKRQ, rVRQ, rRRQ and rPRQ spanning residue 136. PMCA amplification of scrapie VRQ/ARQ prion (PG1361/05) in a VRQ/VRQ substrate was carried out in the absence or presence of a rPrP peptides (all in triplicate, OvV, OvP, OvR and OvK are peptides from VRQ, PRQ, RRQ and KRQ, respectively). Product was analysed by dot blot (each sample side by side in duplicate). Peptides were composed of amino acid residues 122-139 (added to 50 μM) or 112-144 (usually added to 1 μM). For all peptides covering residues 112-144, the no inhibition control was carried out in the equivalent dilution of the peptide carrier solvent, 80% (v/v) acetonitrile (N.B. the solvent present with 50 μM peptide inhibited amplification as seen for OvR*). For

OvV122-139 the no inhibition control was also carried out in the equivalent dilution of the peptide carrier solvent, 18% (v/v) acetonitrile + 2% (v/v) formic acid. All other no inhibition controls were in PBS as the peptides were dissolved in water. Analysis of dot blot images by densitometry measured the PrP^{Sc} signal compared to the corresponding no inhibition control (bar graphs, N.B. bar graphs for OvR peptides are shown in Figure 3). Data was analysed using one-way ANOVA at the concentrations used versus the relevant no inhibition control. This used Dunnett's multiple comparison test for OvP and OvK 122-139, or Šidák's multiple comparison test for all 112-144 peptides and OvV 122-139. No peptide significantly inhibited prion replication. PrP^{C}, brain PMCA substrate used as a PK-digestion control.

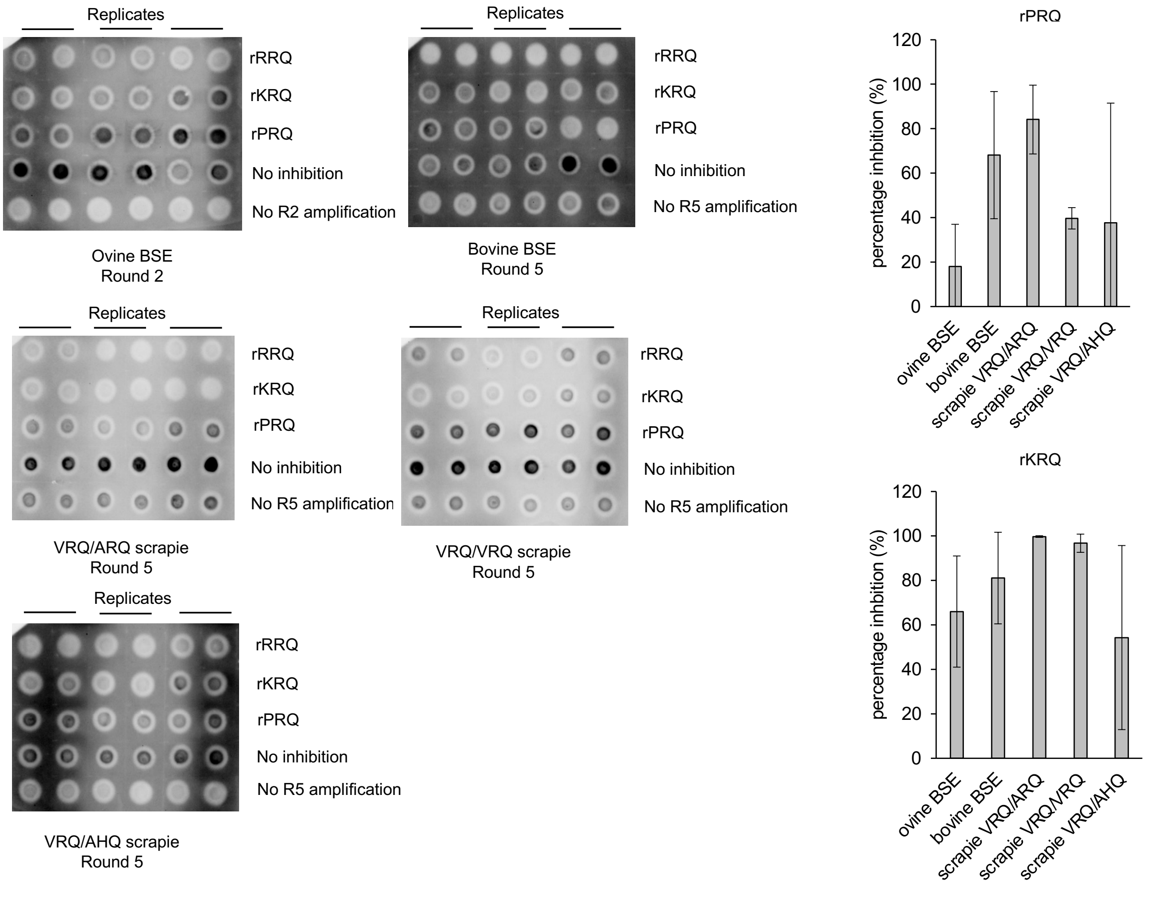

Figure S4. Inhibition of the replication of ruminant prion strains/isolates with 136 variants of rPrP. rRRQ, rPRQ and rKRQ were added (as indicated) into each round of PMCA at 50 nM. PMCA amplification in the absence of any rPrP was carried out as a no inhibition control for comparison. Three replicate PMCA reactions were carried out and each analysed in duplicate by dot blot. Ovine BSE genotype ARQ/ARQ was amplified in 2 rounds using alternating ARQ/ARQ and AHQ/AHQ substrates. Bovine BSE was amplified in bovine brain substrate, and ovine scrapie isolates with VRQ/ARQ, VRQ/VRQ and VRQ/AHQ genotypes amplified in ovine VRQ/VRQ brain substrate; all amplified over 5 rounds and analysed on the same blot with their corresponding no inhibition control and background signal (no amplification in the final round of sPMCA). Dot blot images were analysed by densitometry and the PrP^{Sc} signal above background (samples with no PMCA amplification carried out in the final round of sPMCA) were expressed as a percentage of the no inhibition control. Three replicate PMCA reactions were carried out and each analysed in duplicate by dot blot, SD for the average signal for each of the triplicates are shown (Graphs above for rKRQ and rPRQ and Figure 2 for rRRQ). rRRQ consistently showed high level of inhibition of

prion replication compared to the other inhibitors; it demonstrated 82*, 98*, 99*, 90* and 82*% inhibition for ovine BSE, bovine BSE, VRQ/ARQ, VRQ/VRQ and VRQ/AHQ scrapie, respectively. rKRQ inhibited these strains by 66*, 81, 100*, 97* and 54%, respectively; and rPRQ by 18, 68, 84*, 40 and 38%, respectively). Inhibition reactions marked with an asterisk were statistically significant when analysing data for each rPrP inhibiting the different sPMCA reactions by comparing each amplification +/- inhibitor and applying a one-way ANOVA with Šidák's multiple comparison test.

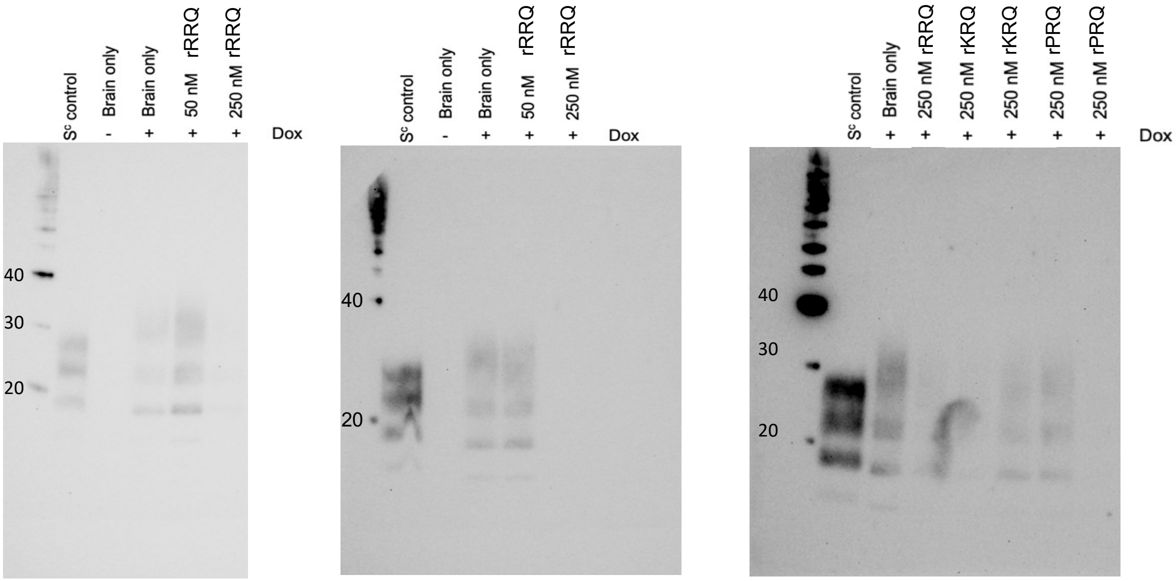

Figure S5. Inhibition of scrapie infection of cells with rPrPs. Rov9 cells expressing ovine VRQ/VRQ PrP^{C} (+ Dox) were inoculated with SSBP/1 scrapie brain homogenate with or without the presence of rRRQ (at 50 or 250 nM, as indicated), rKRQ or rPRQ (both at 250 nM). After a single passage, cell lysate containing 500 µg of total protein was analysed for PrP^{Sc} levels by western blot. Cells without induction of VRQ/VRQ expression (- Dox, brain only) were included in the first two repeats to demonstrate no PrP^{Sc} inoculum was detected in the analysed samples. Uninhibited cell infections were carried out in the absence of any rPrP (+ Dox, brain only). The experiment was repeated twice with inhibitor at 50 nM (rRRQ) and two (rPRQ and rKRQ) or three (rRRQ) times with inhibitor at 250 nM, and western blots are shown. SSBP/1 brain (S^{C} control) was digested with 100 µg/ml PK and the equivalent of 7.5 µl of 10% (w/v) brain was analysed as a blotting control. Protein molecular weight markers are indicated (kDa).

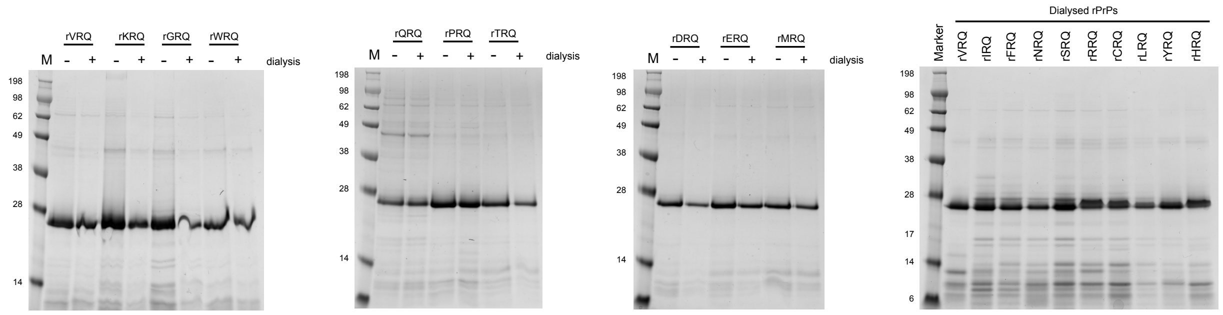

rPrP 136 residue	purity (%)	rPrP 136 residue	purity (%)	rPrP 136 residue	purity (%)	rPrP 136 residue	purity (%)	rPrP 136 residue	purity (%)
V	90	Q	76	F	79	C	71	D	96
K	93	P	83	N	71	L	82	E	93
G	84	T	90	S	71	Y	83	M	81
W	88	I	83	R	71	H	78		

Figure S6. Purity estimations for rPrPs. Following affinity purification, fractions were checked for purity and the purest fractions were pooled. Samples were stored at -80ºC with 20 % (w/v) sucrose. Before rPrP was used as an inhibitor in PMCA or cell infection experiments, imidazole and sucrose were removed by two rounds of dialysis against PBS. The dialysed protein concentration was then determined by Bradford assay and purity estimated by SDS-PAGE analysis (top panels; where samples before and after dialysis were analysed this is indicated, other samples were analysed after dialysis). Purity was estimated by densitometry analysis and was between 71 and 96% (table).

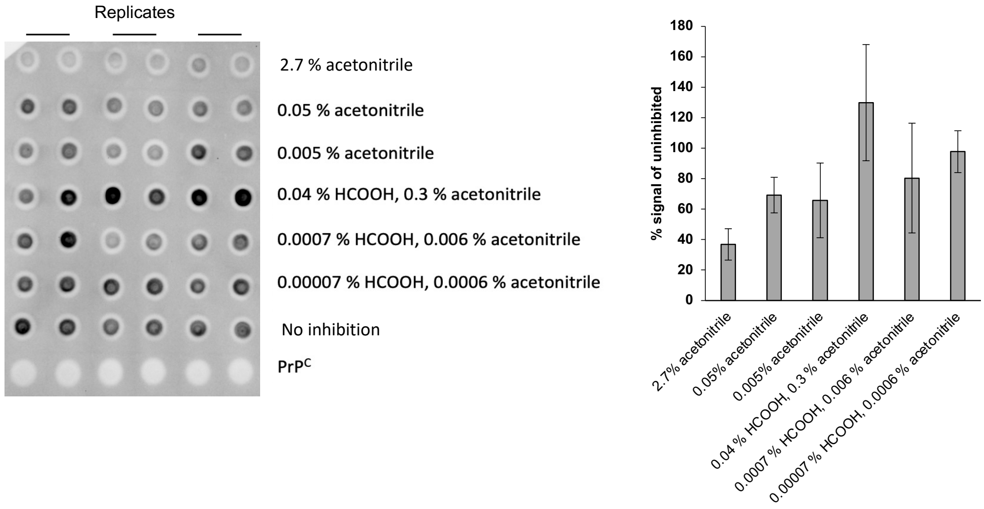

Figure S7. The effects of peptide carrier solvents on prion amplification. For the analysis of PrP peptides as inhibitors, it was first established whether the carrier solvent had any effects on PMCA. The indicated concentrations of solvent (equivalent to that present when adding 50 μM, 1 μM and 100 nM peptide to PMCA reactions), were added into PMCA reactions amplifying ARQ/VRQ PG1361/05. After 1 round of PMCA, 2.5 μl of PK digested products were analysed by dot blot in duplicate. PrP^C, negative brain homogenate to test PK digestion efficiency. Acetonitrile (at concentrations shown) significantly reduced prion amplification compared to the uninhibited control (in PBS). The presence of HCOOH with acetonitrile (at concentrations shown) did not have a significant effect on prion amplification. Analysis of the densitometry data was by one-way ANOVA with Dunnett's multiple comparison test.
